# An open-label study to evaluate a single-dose of cinacalcet in pediatric dialysis subjects

**DOI:** 10.1007/s00467-012-2186-9

**Published:** 2012-05-26

**Authors:** Desmond Padhi, Craig B. Langman, Sahar Fathallah-Shaykh, Bradley A. Warady, Isidro B. Salusky, Edward Lee, Christine Wang, Edward Posvar

**Affiliations:** 1 Early Development, Amgen Inc., One Amgen Center Dr., M/S 38-3-A, Thousand Oaks, CA 91320 USA; 2 Feinberg School of Medicine, Children’s Memorial Hospital, Northwestern University, Chicago, IL USA; 3 Division of Pediatric Nephrology, University of Alabama at Birmingham, Birmingham, AL USA; 4 Children’s Mercy Hospitals and Clinics, University of Missouri–Kansas City School of Medicine, Kansas City, MO USA; 5 Division of Pediatric Nephrology, Department of Pediatrics, David Geffen School of Medicine at UCLA, Los Angeles, CA USA; 6 Metabolic Disorders, Amgen Inc., Thousand Oaks, CA USA

**Keywords:** Hyperparathyroidism, CKD–MBD, Hemodialysis, End-stage renal disease, Pharmacokinetics, Pharmacodynamics, Secondary

## Abstract

**Background:**

There is limited knowledge of the effectiveness and safety profile of cinacalcet in pediatric patients with secondary hyperparathyroidism (sHPT) treated with dialysis.

**Methods:**

This was an open-label, single-dose study conducted on 12 pediatric subjects with chronic kidney disease treated with dialysis. Subjects were stratified by four age cohorts and given a single 15-mg oral dose of cinacalcet. Multiple blood samples were collected up to 72 h post-dose for the assessment of serum calcium (Ca), serum intact parathyroid hormone (iPTH), and plasma cinacalcet concentrations.

**Results:**

Overall, cinacalcet was well tolerated with no serious adverse events. Mean (standard deviation) percentage change in serum Ca over the first 12 h post-dose was −2.93 % (5.70 %) with a nadir of −4.34 % (6.04 %) at 8 h; Ca values returned to baseline by 48 h post-dose. Mean percentage change in iPTH over the first 12 h post-dose was 57.94 % (71.82 %) with a nadir of −35.65 % (55.82 %) at 2 h. There was an inverse relationship between peak serum Ca concentration and body surface area (BSA) (*r* ^2^ = 0.41), although no relationship was found between area under the curve and age or BSA.

**Conclusions:**

These data support future analysis to determine the therapeutic starting dose of cinacalcet for pediatric patients with sHPT on dialysis.

## Introduction

Pediatric and adult patients with chronic kidney disease (CKD) are subject to a unique constellation of signs and symptoms that affect bone and mineral metabolism, termed collectively, CKD–mineral and bone disorders (CKD–MBD). Part of the biochemical picture in CKD–MBD includes elevated blood levels of fibroblast growth factor 23 (FGF23) and reduced blood levels of calcitriol. Markedly high levels of FGF23 in combination with parameters of abnormal mineral metabolism are associated with progressive increases in serum intact parathyroid hormone (PTH), referred to as secondary hyperparathyroidism (sHPT).

The hyperphosphatemia and hypocalcemia seen in CKD–MBD worsens sHPT, a disease associated with bone disease as well as systemic cardiovascular and cutaneous toxicities [1]. Although therapies for sHPT include lowering serum phosphorus (P) through dietary restrictions and the use of phosphate binders, supplementation of calcium (Ca), vitamin D analogs, and parathyroidectomy [2], these options have significant limitations associated to dietary restrictions, medication non-adherence, as well as adverse reactions. In patients with end-stage renal disease (ESRD), vitamin D sterols are effective in the short term in lowering the serum concentration of PTH through both direct and indirect mechanisms [3, 4]. However, the efficacy of vitamin D sterols in suppressing iPTH may be reduced due to their potential to raise serum Ca and P levels.

The calcimimetic Sensipar®/Mimpara® (cinacalcet; Amgen, Thousand Oaks, CA) is an addition to existing dialysis therapies, designed to treat sHPT [5–8]. Cinacalcet belongs to a family of small organic allosteric activators of the G-protein-coupled calcium sensing receptor (CaSR) in the parathyroid glands and other tissues [9]. It increases the sensitivity of extracellular CaSR to extracellular Ca. Activation of CaSR decreases both the *synthesis* and *secretion* of PTH [10] and subsequently decreases serum Ca and P, as well as the Ca × P product in adult dialysis patients. In such patients with sHPT on hemodialysis, cinacalcet has been shown to be effective in lowering PTH levels to clinically acceptable levels that satisfy biochemical target ranges defined by the Kidney Disease Outcomes Quality Initiative (K-DOQI)^TM^ for disease management [5, 7, 8, 11–13]. Cinacalcet doses are adjusted based on response to PTH levels.

Approximately 80 % of pediatric patients with CKD and on dialysis are treated for renal osteodystrophy. There is little evidence that parathyroid gland function differs in children and adolescents with ESRD compared to adults [14]. Thus, there is reason to believe that pediatric dialysis patients with sHPT will also respond to treatment with cinacalcet. To date, there are no published randomized controlled studies evaluating the safety and efficacy of cinacalcet in the pediatric dialysis population. However, several retrospective observational studies have demonstrated that PTH levels decrease following the initiation of cinacalcet treatment, including that of Silverstein et al. [15] who found decreased PTH levels within the first month of initiating cinacalcet treatment, with no change in Ca, P, or Ca × P after 3 months in nine pediatric dialysis patients, while Muscheites et al. [16] reported statistically significant and clinically relevant decreases for PTH, Ca, P, and Ca × P within a 4-week treatment period. Although these findings are consistent with the literature on cinacalcet in the adult populations [17], both studies were based on adult dosing guidelines and illustrate the need to further investigate cinacalcet use prospectively in the pediatric dialysis population.

Limited therapeutic options highlight an unmet need for a more effective therapy for sHPT in pediatric patients on dialysis. Thus, the study reported here was designed to evaluate the safety, tolerability, pharmacokinetics, and pharmacodynamics of a 15-mg dose of cinacalcet in pediatric subjects with sHPT treated with hemodialysis.

## Methods

This was a single-dose, open-label study to evaluate the safety, tolerability, pharmacokinetics, and pharmacodynamics of cinacalcet in pediatric subjects with CKD on dialysis. The ethics committees of each study center reviewed and approved the study protocol and the consent forms prior to the investigators enrolling subjects. Twelve subjects were enrolled, and all subjects continued their regular therapy for management of sHPT (e.g., phosphate binder, vitamin D sterol). Oral or intravenous Ca therapy and alteration of the dialysate calcium concentration was allowed for serum Ca <1.87 mmol/L (7.5 mg/dL) or signs or symptoms of hypocalcemia subsequent to the study drug administration.

Seven girls and five boys aged 6–17 years on dialysis for at least 1 month at the time of screening were enrolled in the study. Written informed consent was obtained from the parent or guardian for all subjects; additionally, written assent was obtained from subjects ≥12 years of age. Subjects were eligible to participate if they had serum Ca level of ≥8.4 mg/dL at the time of the baseline visit and had not received any cinacalcet therapy for at least 2 weeks before Day 1 dosing.

To ensure subjects were enrolled across the entire age range of 6–17 years, three subjects were required to be enrolled into each of the following age-cohorts: 6–8, 9–11, 12–14, and 15–17 years. After a minimum 8-h fast, each subject received a single oral 15-mg dose of cinacalcet (½ of a 30-mg tablet, weighed with a Mettler balance) with 90 mL (3 oz) of water. Subjects continued to fast for 4 h after treatment administration, and they were followed for 72 h after dosing.

### Rationale for dosage selection

In vitro and in vivo studies have demonstrated that cinacalcet is extensively metabolized by multiple cytochrome P450 (CYP) enzymes, including CYP3A4, CYP2D6, and CYP1A2 [18]. The capacity to metabolize drugs in children varies throughout development of CYP enzymes and is completed by approximately 6 years of age. Therefore, the difference in pharmacokinetic properties of cinacalcet for adults and pediatric patients older than 6 years is likely to be due to differences in body surface area (BSA). Based on BSA, a 6-year-old child should receive approximately 50 % of an adult dose. The recommended starting dose for cinacalcet in adults is 30 mg; therefore, a dose of 15 mg was selected for this study.

### Sample collection

Blood samples for safety assessments were collected at screening, baseline, and on study Day 4. Blood samples for pharmacokinetic analysis were collected at predose and at 0.5, 1, 2, 3, 4, 5, 6, 8, 12, 24, 48, and 72 h post-dose, and those for pharmacodynamic (Ca and iPTH) analysis were obtained at predose and at 2, 4, 8, 12, 24, 48, and 72 h post-dose.

### Planned statistical analysis

All subjects who received cinacalcet were included in the safety, pharmacokinetics, and pharmacodynamics analyses. Since this was a descriptive study, the sample size of three subjects per age-cohort was based on practical considerations; no formal analyses for between-cohort comparisons were conducted.

The safety profile assessment was based on adverse events, vital signs, clinical laboratory measurements, electrocardiogram, and physical examinations. An adverse event was defined as an undesirable medical sign or symptom or worsening of a pre-existing medical condition present at baseline that occurred after initiation of the investigational product. A serious adverse event included any event deemed fatal or life threatening, an event that required or prolonged in-patient hospitalization, or a persistent, significant disability or incapacity, congenital anomaly, or birth defect, as well as any event that may have resulted in urgent investigation.

Plasma cinacalcet concentration–time data were analyzed by non-compartmental methods using WinNonlin Enterprise (ver. 5.1.1; Pharsight Corp, Mountain View, CA). The serum peak concentration (C_max_) after dosing was identified by inspection of the data, and the corresponding time to reach C_max_ (t_max_) was recorded. Plasma cinacalcet concentration versus time was plotted on a semi-log-scale, and the data points that described the terminal log-linear segment of the elimination phase were identified. Whenever possible, a linear regression of the log-transformed terminal data points versus time was used to estimate the terminal rate constant β. The terminal half-life, t_1/sβ_ was calculated as 0.693/β. The area under the concentration–time curve from zero to the last measurable concentration (AUC_0-t_) was calculated by the linear trapezoidal method. The AUC from the time of the last measurable concentration to infinity (AUC_t-inf_) was calculated by dividing the predicted concentration at the time of the last measurable concentration by β. The AUC from time zero to infinity, AUC_o-inf_, was calculated by the summation of AUC_0-t_ and AUC_t-inf_. AUC_0-inf_ was not reported when AUC_t-inf_ exceeded 20 % of the total AUC. The apparent oral clearance (CL/F) was calculated as dose/AUC_0-inf_.

## Results

### Study subjects

Table 1 shows the baseline demographics of the 12 pediatric subjects with mean (SD) age of 11.3 (3.7) years receiving chronic hemodialysis or peritoneal dialysis that were enrolled in this single dose study. All subjects were included in all analysis sets.

**Table 1:** Baseline demographics

Patient baseline demographics	15 mg cinacalcet (*n* = 12)
Female, *n* (%)	7 (58)
Hispanic or Latino, *n* (%)	6 (50)
Black or African American, n (%)	3 (25)
White or Caucasian, *n* (%)	3 (25)
Age (years), mean (SD)	11.33 (3.68)
Height (cm), mean (SD)	136.13 (20.53)
Weight (kg), mean (SD)	38.38 (15.67)
BMI (kg/m^2^), mean (SD)	19.85 (3.53)

BMI, Body mass index; SD, standard deviation

### Safety

Hypocalcemia (Ca <2.23 mmol/L), a documented effect of cinacalcet in studies with adults, was reported in 50 % (*n* = 6) of subjects, with a range of 2.00–2.22 mmol/L. Other reported (*n* = 2, 17 %) adverse events were mild to moderate in severity and assessed by the investigators to be unrelated to the study drug. Bacteremia and pyrexia (with normal Ca levels) were reported for one subject (age 11 years) 48 h post-dose. One subject (age 15 years) with a 6-month history of ESRD had an adverse event of QT interval prolongation within 72 h post-dose, which the investigator related to prior trauma. Subsequent electrocardiograms performed 2 and 6 weeks after the date of onset showed a prolonged QTc interval and non-specific ST segment changes. After the 3-month follow-up visit the subject was referred to a cardiologist who documented a normal QTc during assessment. Based on the cardiologist’s report and the subject’s negative family history for prolonged QT interval prolongation, the investigator considered the adverse event to be unrelated to the investigational product. Neither of these two subjects withdrew from the study due to the adverse event, and they reported no serious adverse events.

Of the 12 subjects, six had decreases in serum Ca below the lower limit of normal without any associated symptoms. The low Ca values for these subjects ranged from 2.00 to 2.20 mmol/L up to 12 h post-dose. No clinically significant trends were observed for the other serum chemistry, hematology, or urinalysis laboratory assessments.

### Pharmacokinetics

**Fig. 1:**
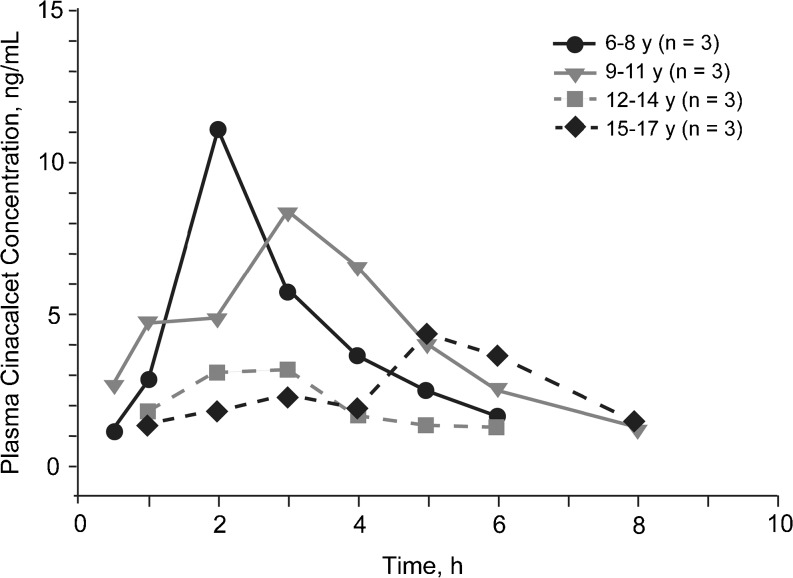
Mean plasma cinacalcet concentration–time profiles following oral administration of 15-mg cinacalcet

**Fig. 2:**
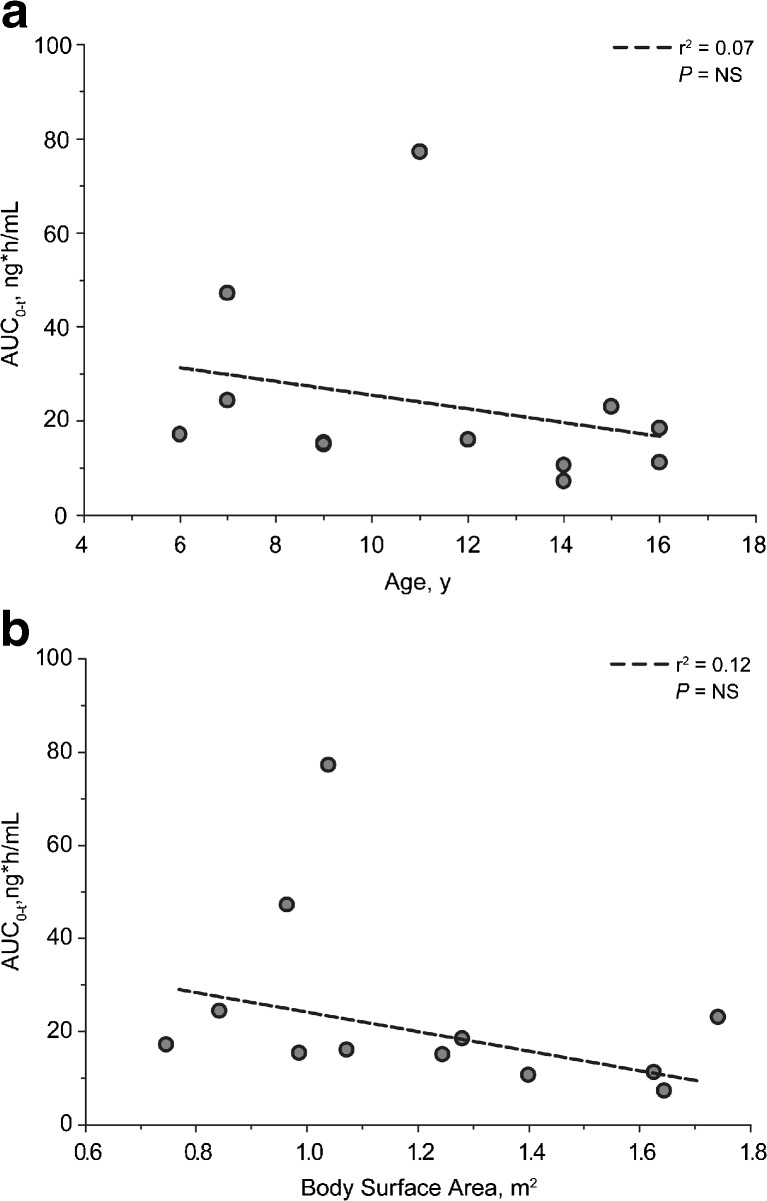
**a** Area under the time–concentration curve from zero to the last measurable concentration (*AUC*_*0-t*_) by age for individual subjects. **b** AUC_0-t_ by body surface area (BSA) for all subjects. *NS* Not significant

**Fig. 3:**
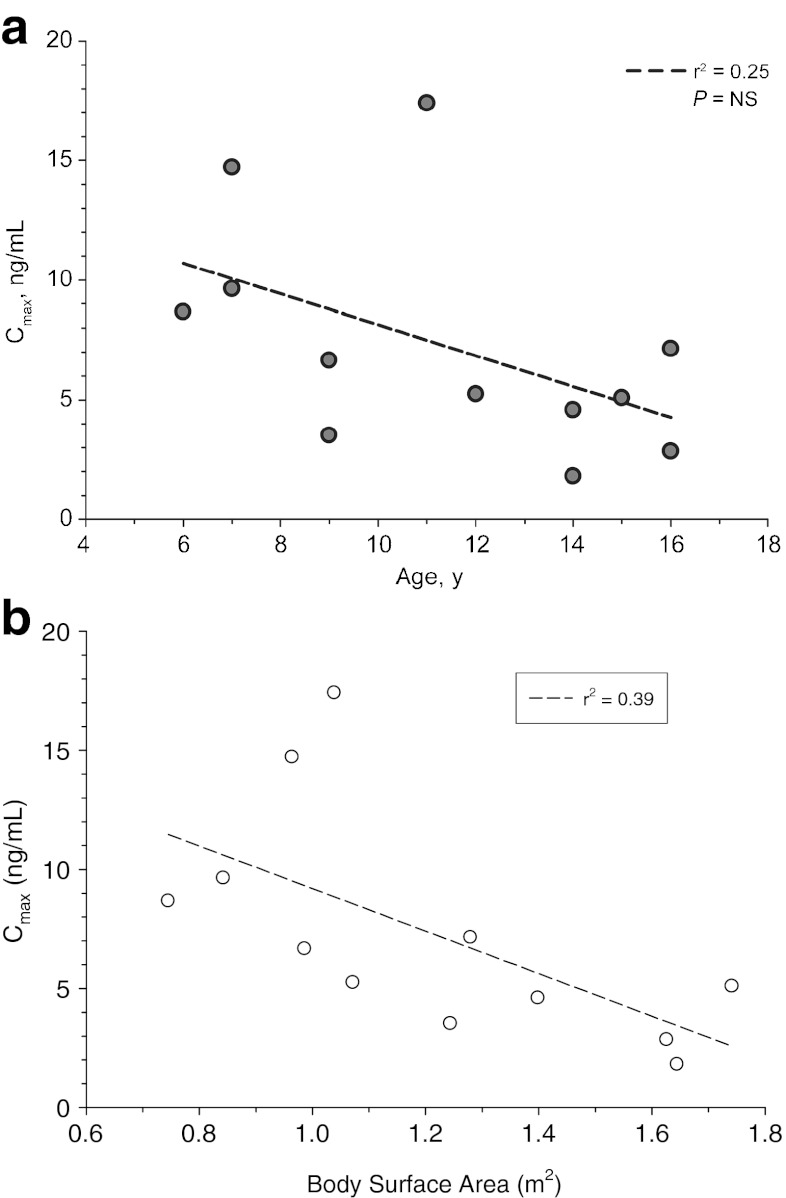
**a** Serum peak cinacelcet concentration (*C*_*max*_) by age for individual subjects. **b** C_max_ by BSA for individual subjects. *NS* Not significant

Mean plasma cinacalcet concentration–time profiles by age-cohort are presented in Fig. 1. For the four age cohorts (*n* = 3 in each), namely, 6–8 years, 9–11 years, 12–14 years, and 15–17 years, the values for the pharmacokinetic parameters were: AUC_0-t_ [h ng/mL; mean (SD)], 29.5 (15.6), 35.9 (35.8), 11.3 (4.4), and 17.5 (5.9), respectively; C_max_ [mg/mL; mean (SD)], 11.0 (3.2), 9.19 (7.28), 3.87 (1.82), and 5.01 (2.15), respectively; t_max_ [h; median (min–max)], 2.0 (2.0–2.0), 3.0 (2.0–4.0), 3.0 (1.0–3.0), and 3.0 (2.0–5.0), respectively. Age and BSA versus AUC_0-t_ , and age and BSA versus C_max_ are presented graphically in Figs. 2a, b and 3a, b, respectively.

Mean exposure (AUC_0-t_ and C_max_) in the four age-cohorts did not consistently increase with decreasing age. However, a trend of greater exposure was observed in younger subjects as well as a trend of increasing exposure with decreasing BSA. Values for AUC_0-inf_, t_1/2β_, and CL/F were not calculable in most subjects.

### Pharmacodynamics

**Fig. 4:**
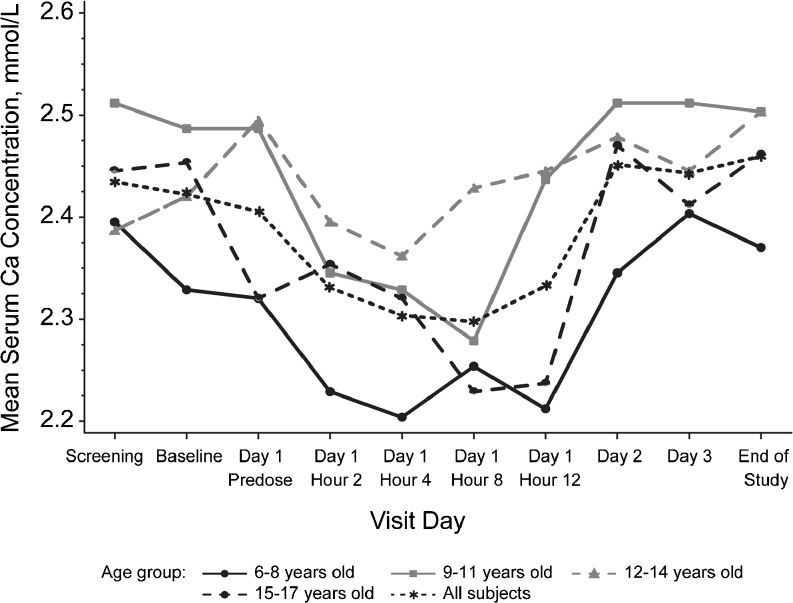
Mean serum calcium (*Ca*) concentrations by time for each age-cohort. For all subjects, the mean ± standard deviation maximum decrease was 4.3 ± 6.0 % 8 h post-dose; Ca returned to the baseline level by Day 2. The overall patterns of change from baseline were similar for all age-cohorts

**Fig. 5:**
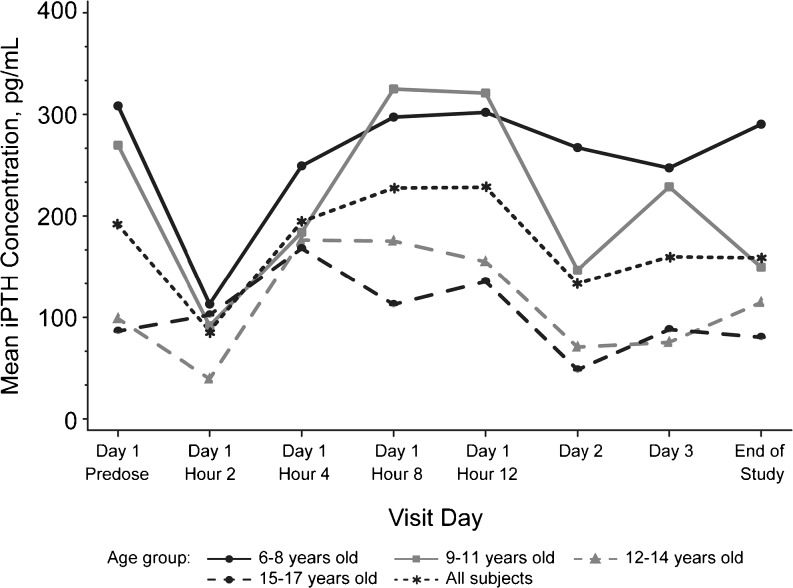
Mean serum intact parathyroid hormone (*iPTH*) concentrations by time for each age-cohort. Maximum mean ± standard deviation decrease was 35.7 ± 55.8 % at 2 h post-dose; PTH returned to baseline level by Day 2. The observed patterns of change from baseline were similar for each age-cohort

Consistent with the pharmacologic action of cinacalcet, mean (SD) percentage changes from baseline in serum Ca (mmol/L) concentration were observed from 2 [−2.88 (5.40)] to 12 h [−2.93 (5.70)] post-dose; returning to baseline levels by Day 2 (Fig. 4; Table 2). Mean (SD) percentage changes in serum iPTH (pg/mL) concentrations decreased −35.65 (55.82) from baseline values up to 2 h post-dose, increased to above baseline between 4 [24.10 (76.14)] and 12 h [57.94 (71.82)] post-dose, and returned to baseline levels by Day 2 (Fig. 5; Table 3). The observed patterns of changes from baseline in mean serum Ca and iPTH concentrations was similar for each age cohort (Figs. 4, 5); the small sample size per age-cohort precluded formal analysis.

**Table 2:** Mean changes from baseline over time in serum calcium level for each age-cohort

Time	Age-cohort				All subjects (*n* = 12)
	6–8 years (*n* = 3)	9–11 years (*n *= 3)	12–14 years (*n* = 3)	15–17 years (*n* = 3)	
Day 1, hour 2					
Absolute (mmol/L)	−0.09 (0.03)	−0.14 (0.13)	−0.10 (0.20)	0.03 (0.12)	−0.07 (0.13)
Percentage	−3.99 (1.47)	−5.51 (4.63)	−3.81 (7.58)	1.80 (5.76)	−2.88 (5.40)
Day 1, hour 4					
Absolute (mmol/L)	−0.12 (0.07)	−0.16 (0.16)	−0.13 (0.20)	0.00 (0.26)	−0.10 (0.17)
Percentage	−5.13 (3.43)	−6.13 (6.08)	−5.18 (7.71)	0.78 (11.99)	−3.92 (7.35)
Day 1, hour 8					
Absolute (mmol/L)	−0.07 (0.10)	−0.21 (0.12)	−0.07 (0.25)	−0.09 (0.12)	−0.11 (0.15)
Percentage	−2.85 (4.36)	−8.25 (4.46)	−2.63 (10.13)	−3.63 (5.28)	−4.34 (6.04)
Day 1, hour 12					
Absolute (mmol/L)	−0.11 (0.09)	−0.05 (0.20)	−0.05 (0.22)	−0.08 (0.10)	−0.07 (0.14)
Percentage	−4.63 (4.01)	−1.73 (7.80)	−1.98 (8.67)	−3.37 (4.32)	−2.93 (5.70)
Day 2					
Absolute (mmol/L)	0.02 (0.02)	0.02 (0.14)	−0.02 (0.25)	0.15 (0.09)	0.05 (0.14)
Percentage	1.12 (1.14)	1.22 (5.51)	−0.54 (9.86)	6.74 (4.60)	2.13 (5.96)
Day 3					
Absolute (mmol/L)	0.08 (0.08)	0.02 (0.18)	−0.05 (0.19)	0.09 (0.10)	0.04 (0.14)
Percentage	3.72 (3.45)	1.29 (7.10)	−1.86 (7.30)	3.97 (4.37)	1.78 (5.52)
End of study					
Absolute (mmol/L)	0.05 (0.10)	0.02 (0.32)	0.01 (0.18)	0.14 (0.13)	0.05 (0.18)
Percentage	2.26 (4.27)	1.19 (12.54)	0.55 (7.10)	6.42 (6.23)	2.60 (7.34)

Data are presented as the mean, with the standard deviation given in parenthesis

**Table 3:** Mean changes from baseline over time in serum intact parathyroid hormone level for each age-cohort

Time	Age-cohort				All subjects (*n* = 12)
	6–8 years (*n *= 3)	9–11 years (*n* = 3)	12–14 years (*n* = 3)	15–17 years (*n* = 3)	
Day 1, hour 2					
Absolute (pg/mL)	−195.33 (159.07)	−178.00 (164.08)	−59.33 (42.36)	16.00 (47.57)	−104.17 (135.92)
Percentage	−63.83 (9.35)	−14.03 (99.53)	−55.26 (20.34)	−9.49 (56.84)	−35.65 (55.82)
Day 1, hour 4					
Absolute (pg/mL)	−59.00 (101.53)	−85.67 (95.47)	77.67 (87.64)	81.67 (151.96)	3.67 (124.61)
Percentage	9.09 (66.66)	−20.76 (45.04)	80.76 (101.55)	27.32 (83.12)	24.10 (76.14)
Day 1, hour 8					
Absolute (pg/mL)	−11.00 (89.40)	55.33 (47.65)	76.67 (76.96)	26.33 (29.19)	36.83 (65.41)
Percentage	10.36 (29.83)	79.50 (104.36)	66.84 (70.91)	42.44 (26.78)	49.78 (62.81)
Day 1, hour 12					
Absolute (pg/mL)	−6.33 (87.32)	51.33 (69.87)	56.33 (92.64)	49.00 (57.19)	37.58 (71.68)
Percentage	25.29 (54.91)	96.80 (126.19)	41.18 (67.17)	68.46 (21.63)	57.94 (71.82)
Day 2					
Absolute (pg/mL)	−41.00 (104.37)	−123.00 (180.59)	−28.00 (4.58)	−38.00 (65.05)	−57.50 (101.33)
Percentage	−6.03 (22.17)	26.84 (117.42)	−31.55 (13.24)	−15.32 (36.59)	−6.52 (58.03)
Day 3					
Absolute (pg/mL)	−61.00 (114.01)	−41.00 (47.79)	−23.33 (11.02)	1.67 (7.23)	−30.92 (58.22)
Percentage	−18.62 (22.40)	61.88 (136.94)	−23.38 (4.68)	−7.25 (12.37)	3.16 (69.46)
End of study					
Absolute (pg/mL)	−18.00 (68.35)	−120.00 (139.57)	16.00 (35.34)	−6.00 (15.87)	−32.00 (87.42)
Percentage	−7.45 (17.58)	211.79 (457.53)	16.38 (41.31)	9.58 (26.94)	57.58 (217.46)

Data are presented as the mean, with the standard deviation given in parenthesis

## Discussion

In this open-label study, we evaluated the safety, tolerability, pharmacokinetics, and pharmacodynamics of a single, oral 15-mg dose of cinacalcet in pediatric subjects 6–17 years of age with CKD who were on dialysis upon entry into the study. No serious adverse events developed as a result of hypocalcemia. All adverse events were mild to moderate in severity. The adverse events reported by the two subjects, bacteremia with pyrexia in one subject and a prolonged QTc interval in the second subject, were considered by the investigators to be unrelated to treatment with cinacalcet. Overall, cinacalcet was well tolerated.

The mean pharmacokinetics exposure did not consistently increase with decreasing age; however, an overall trend of greater exposure in younger subjects was observed, which may be related to the lower body weight and BSA in younger subjects compared with older subjects. The mean AUC_0-t_ and C_max_ values following a single 15-mg dose of cinacalcet for the combined cohorts in this study were within approximately 30 % of values observed following a single 30-mg dose (one 30-mg tablet) of cinacalcet administered to healthy adult subjects in a previous study (Amgen study 20060133; data on file).

The patterns of changes in serum Ca and iPTH concentration from baseline were similar between cohorts and within the expected ranges for patients with CKD. These findings are consistent with published data, which demonstrate a reduction in plasma iPTH levels as well as serum Ca and phosphorus levels for adult patients on hemodialysis following the administration of cinacalcet [5, 8, 19, 20]. Furthermore, results from small observational studies suggest that the administration of cinacalcet when PTH levels are between 300–500 pg/mL or 501–800 pg/mL decreases PTH to <300 pg/mL within 16–28 weeks of therapy [11] (Amgen; data on file). Achieving therapeutic target ranges is important in decreasing the risk of other bone and mineral comorbidities associated with sHPT that when left untreated can lead to increased rates of hospitalization.

Although in combination with PTH level dialysis vintage may affect biochemical responses to cinacalcet, Padhi et al. previously reported that the use of dialysis (hemodialysis and peritoneal dialysis) does not affect the pharmacokinetics or pharmacodynamics of cinacalcet, as it is a lipophilic drug that is approximately 96 % protein bound and not dialyzable [21]. Their study showed that baseline PTH levels increased with decreased renal function, while changes in Ca were not consistent from baseline to nadir and at peak concentrations. Therefore, the dose in the present study, based primarily on the BSA of subjects, was appropriately determined and did not need further modification based on dialysis.

These data are important since they provide the first formal pharmacokinetic and pharmacodynamic data related to the use of cinacalcet in children and support further investigation of cinacalcet to establish the appropriate starting dose for the treatment of sHPT in pediatric patients receiving dialysis. These data will now serve as the basis for determining drug dosing in prospective clinical trials designed to evaluate the safety and efficacy of cinacalcet in the pediatric dialysis population with sHPT.

### Limitations of current study

This was a small, open-label study to evaluate the safety and describe the administration of a single-dose of cinacalcet in pediatric dialysis patients with sHPT. Due to the sample size no formal statistical comparisons were made in this study. Dialysis vintage was not collected; however, it was reportedly brief compared to adults since most pediatric patients are transplanted or return from transplant. Although the results from our study are consistent with observations in adults from the same population, the current analysis precludes drawing strong conclusions at this time. However, future studies of pediatric patients with a larger sample size will likely yield comparable results to the adult population.
